# Receptor Proteins in Selective Autophagy

**DOI:** 10.1155/2012/673290

**Published:** 2012-03-20

**Authors:** Christian Behrends, Simone Fulda

**Affiliations:** ^1^Institute of Biochemistry II, Goethe University School of Medicine, 60590 Frankfurt, Germany; ^2^Institute for Experimental Cancer Research in Pediatrics, Goethe University, 60590 Frankfurt, Germany

## Abstract

Autophagy has long been thought to be an essential but unselective bulk degradation pathway. However, increasing evidence suggests selective autophagosomal turnover of a broad range of substrates. Bifunctional autophagy receptors play a key role in selective autophagy by tethering cargo to the site of autophagosomal engulfment. While the identity of molecular components involved in selective autophagy has been revealed at least to some extent, we are only beginning to understand how selectivity is achieved in this process. Here, we summarize the mechanistic and structural basis of receptor-mediated selective autophagy.

## 1. Introduction

**Figure 1:**
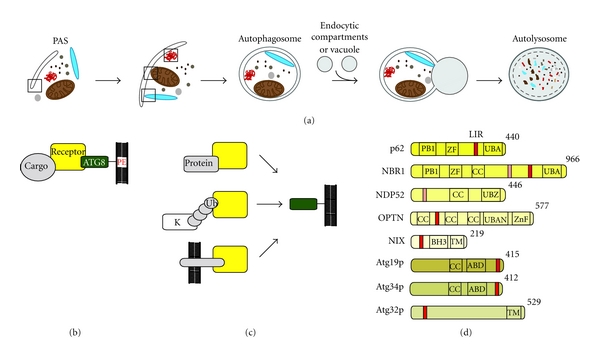
(a) Overview of selective autophagy. Boxes indicate localization of ATG8 and autophagy receptor proteins. (b) Scheme of autophagy receptor function. (c) Different cargo-binding concepts of autophagy receptors. (d) Domain architecture of the known characterized autophagy receptors.

Macroautophagy or bulk autophagy (referred to as autophagy in the text) is an evolutionarily highly conserved program for sequestration and transport of macromolecules and organelles to the vacuole or lysosomal compartment where they are degraded [1–4]. This form of autophagy is considered to be a rather unselective process for bulk degradation of cellular constituents that serve to recycle macromolecules to maintain cellular homeostasis and energy balance and to provide new building blocks for anabolic processes under deprivation of nutrition [5]. In addition, autophagy represents a quality control mechanism to clear damaged or surplus organelles and aggregated or misfolded proteins, respectively [6]. Autophagy is engaged by the formation of the isolation membrane or phagophore, a double membrane that enlarges and wraps around cytosolic cargo yielding a closed multilamellar vesicular structure, coined autophagosome. The subsequent fusion of autophagosomes with the vacuole in yeast or with lysosomes in mammalian cells initiates degradation of enclosed cargo by acidic hydrolases (Figure 1(a)). In contrast to bulk autophagy, selective autophagy involves targeted recognition and removal of protein inclusions, organelles or microbes [7]. A set of specific proteins play a pivotal role in both the recognition as well as the delivery of cytoplasmic cargo to the incipient autophagosome for engulfment and ultimately lysosomal degradation [8, 9]. These so-called autophagy receptors mediate simultaneous binding of cytosolic cargo and components of the autophagy machinery (Figure 1(b)). The modular composition of binding domains and motifs in autophagy receptor proteins ensures efficient tethering of cargo to the site of developing and engulfing autophagosomes.

## 2. Cargo Binding Domains in Autophagy Receptors

Autophagy receptors can be grouped based on their specific cargo-binding domains. Fundamental different principals have been employed for the use of these binding domains in selective autophagy ranging from protein-specific interaction domains via posttranslational modification- (PTM-) binding domains to transmembrane domains (Figure 1(c)). While protein-specific interaction domains yield autophagosomal delivery of only a set of very specialized targets, PTM-specific binding domains, namely, ubiquitin-binding domains, allow for autophagy engagement of a huge variety of proteins. Lastly, by the virtue of membrane embedding, autophagy receptors mediate organelles-specific targeting for selective autophagy. 

## 3. Protein-Specific Binding Domains

In yeast, at least two vacuole-resident enzymes, aminopeptidase 1 (Ape1p) and *α*-mannosidase (Ams1p), are selectively and constitutively transported into the vacuole as part of their biosynthesis via an autophagy-like process called cytoplasm to vacuole targeting (Cvt) pathway [10]. Following translation in the cytosol as pro-enzyme (prApe1p), Ape1p oligomerizes into a dodecamer and further assembles with Ams1p and the autophagy receptor Atg19p into large so-called Cvt complexes. Atg19p binds to the propeptide of prApe1p and to Ams1p via its central coiled coil and carboxy-terminal Ams1-binding domain (ABD) (Figure 1(d)), respectively, and is essentially required for recruitment of the Cvt complex to the pre-autophagosomal structure (PAS) prior to vacuolar delivery [10]. The autophagy receptor Atg34p, a recently characterized Atg19p homolog, acts cooperatively with Atg19p in the Cvt pathway [11, 12]. Like Atg19p, Atg34p contains a C-terminal ABD (Figure 1(d)). However, since Atg34p lacks the prApe1p-binding specific coiled coil, Atg34p mediates the delivery of Ams1p to the vacuole but not of prApe1p [12]. The structures of ABD in Atg19p and Atg34p have recently been solved and show an eight *β*-strand-composed immunoglobulin-like fold [12]. Though, the exact Ams1p-binding mechanism by ABD has not been determined in detail yet. Likewise, we do not understand structurally how the coiled coil of Atg19p binds prApe1p. Recently, another biosynthetic enzyme, leucine aminopeptidase III (Lap3p), has been identified as Atg19p-dependent cargo for selective autophagy under starvation conditions [13], indicating that more proteins than previously anticipated might be delivered to the vacuole via forms of selective autophagy. However, whether Atg19p's coiled coil or ABD domain mediates Lap3p binding and whether additional Cvt receptor proteins exist is currently unknown. An intriguing question remains whether mammalian homologues of these enzymes (i.e., LAP3) employ selective autophagy pathways for their lysosome targeting.

## 4. Ubiquitin-Specific Binding Domains

Covalent attachment of ubiquitin to proteins has emerged as a versatile regulatory signal mediating several forms of selective autophagy targeting aggregated proteins (aggrephagy), bacterial pathogens (xenophagy), and damaged mitochondria (mitophagy) [8, 9]. Ubiquitylation occurs through isopeptide bond formation between the *ε*-amino group of a lysine residue in a target protein and the C-terminal carboxyl group of ubiquitin [14, 15]. Proteins can be modified by ubiquitin monomers (monoubiquitylation and multi-monoubiquitylation) or by ubiquitin polymers (polyubiquitylation), in which ubiquitin moieties are most often connected via lysine-mediated isopeptide linkages [16]. Different chain linkage types arise from the fact that all 7 lysine residues in ubiquitin (K6, K11, K27, K29, K33, K48, and K63) as well as the N-terminal methionine serve as ubiquitin acceptor [17, 18]. These diverse ubiquitin signals are decoded by distinct classes of ubiquitin-binding domains [19, 20].

So far, three different ubiquitin-binding domains have been implicated in specific cargo receptors for selective autophagy: ubiquitin-associated (UBA), ubiquitin binding in A20-binding inhibitor of NF-kappa-B (ABIN) and NF-kappa-B essential modulator (NEMO) (UBAN), and ubiquitin-binding zinc finger (UBZ) domains. While the UBA domain is found in p62/SQSTM1 (referred to as p62) and neighbor of BRCA1 (NBR1), UBAN and UBZ domains are found in optineurin (OPTN) and nuclear dot protein 52 (NDP52), respectively (Figure 1(d)). In contrast to the aforementioned autophagy receptors Atg19p and Atg34p, which bind directly to their cargo, this group of ubiquitin-binding domain-containing receptors binds to cargo in an ubiquitin-dependent manner. Thus, implementation of ubiquitin-binding domains in autophagy cargo receptors provides a flexible signal, which allows a much broader range of proteins to be targeted for autophagosomal degradation. So far, a variety of cargos have been discovered, which depend on its ubiquitylation to be efficiently incorporated into autophagosomes, including protein aggregates, mitochondria (via ubiquitylation of outer mitochondrial membrane proteins), and microbes (via ubiquitylation of bacterial membrane proteins or host binding proteins) [7–9]. Notably, though the ubiquitin E3 ligases CHIP and Parkin have been implicated in ubiquitylation of misfolded proteins and damage mitochondria, respectively [21–23], the machineries, which are responsible for targeted ubiquitylation of these distinct autophagosomal substrates, are not clearly defined yet.

Defining the ubiquitin chain linkage preference of ubiquitin-binding domains employed in the known autophagy receptors will be critical to fully understand the molecular basis of ubiquitin-mediated selective autophagy. While it has been established that p62's UBA domain binds both K63 and K48 polyubiquitin chains but with higher affinities to K63 ubiquitin chains [24, 25], the picture is less clear for NBR1. The isolated UBA domain of NBR1 binds to K63 and K48 polyubiquitin chains with a slight preference for K63 ubiquitin chains *in vitro* [26], whereas the chain type specificity of full-length NBR1 has not been determined conclusively. NDP52 follows a similar trend as p62 and NBR1 by preferentially binding to K63 polyubiquitin chains [27]. Finally, OPTN's UBAN domain binds specifically to linear polyubiquitin chains as paradigmatically shown for NEMO [27, 28].

p62 and NBR1 cooperatively mediate aggrephagy [26, 29, 30]. Ubiquitylated proteins are bound via their respective UBA domain and consequently delivered to autophagosome. Besides the C-terminal UBA domain, p62 and NBR1 share common N-terminal Phox and Bem1 (PB1) domains (Figure 1(d)), which mediate homooligomerization of p62 and that drives multimerization of p62 and NBR1 in complex with ubiquitylated proteins, thereby amplifying the engagement of ubiquitylated proteins [31]. Importantly, formation of ubiquitylated protein aggregates required polymerization and ubiquitin binding by p62 and possible NBR1 mediated by UBA and PB1 domains, respectively [29]. Sequestration of misfolded proteins into aggregated inclusions likely shields aberrantly exposed hydrophobic surfaces from harmful interaction with essential cellular proteins and might serve as a sink fueling subsequent autophagosomal or proteasomal degradation [32]. Clearance of p62-driven aggregates depends on constitutive autophagy, since autophagy deficiency by ATG7 depletion causes accumulation of ubiquitylated protein inclusions, which were substantially reduced in ATG7/p62 double knockout cells [33]. Notably, p62 and NBR1 are themselves autophagy substrates, which are continuously degraded along with their bound substrates [26, 29, 30]. Elevated levels of p62 caused by autophagy inhibition have been shown to compromise degradation of proteasome substrates [34]. Thus, shifting the abundance of p62 (and possibly NBR1) might lead to competition with other ubiquitin-binding proteins such as ubiquitin shuttling factors or proteasomal ubiquitin receptors, ultimately causing a nonproductive partitioning of ubiquitylated substrates from proteasomes to p62 aggregates. Notably, K48 and K63 ubiquitin chains together with monoubiquitin have been implicated in the formation of protein inclusion but only K63 chains contributed to autophagic clearance of these aggregates [35]. Furthermore, p62-positive aggregates are commonly detected in neurodegenerative diseases, which are often accompanied by proteasome dysfunction [36]. Though NBR1 and p62 have partially redundant functions, we do not fully understand their individual contribution and requirement for driving aggregate formation in the context of selective autophagy.

 Together with NDP52 and OPTN, p62 participates in the cellular defense mechanism against infection termed xenophagy [27, 37, 38]. Mammalian cells ubiquitylate bacteria that intrude the cytosol or reside in sequestered membrane compartments as part of their protective response thereby marking these microbes for destruction by selective autophagy [3, 4, 38]. Recent studies have shown that clearance of ubiquitylated bacteria is mediated by specific autophagy receptors that facilitate the assembly of an autophagosomal membrane surrounding the bacterial invaders and deliver them to the autophagosomal degradation machinery. This selective removal of invading bacteria by autophagic degradation has been described to protect cells from bacterial colonization [27, 37]. For example, p62 has been implicated in clearance of *Salmonella*. It was reported that p62 is recruited to ubiquitin-decorated *Salmonella* in the cytosol via its UBA domain [38]. Furthermore, NDP52 has recently been described to recognize ubiquitylated *Salmonella* and to restrict their cytosolic growth by destruction via the autophagy pathway [37]. NDP52 binds to ubiquitin-coated bacteria and recruits the TANK-binding kinase 1 (TBK1) via the adaptor proteins Nap1 and Sintbad [37]. In addition, p62 and NDP52 proteins were recently reported to target *Shigella *and *Listeria* to distinct autophagy pathways [39]. Recently, OPTN has been reported to restrict the pathogenic cytosolic growth after bacterial infection with *Salmonella *[27]. As for NDP52, OPTN recruitment to ubiquitylated *Salmonella* required a functional ubiquitin-binding domain. Interestingly, OPTN and NDP52 were reported to localize to common microdomains on ubiquitin-coated bacteria that could be separated from those occupied by p62 [27]. Similarly, NDP52 and p62 were described to localize to non-overlapping microdomains on the surface of ubiquitylated bacteria to target *Salmonella* to the autophagy pathway [40]. Depletion experiments indicated that all three selective autophagy adaptor proteins, that is, NDP52, p62, and OPTN, act in the same pathway to cooperatively drive efficient autophagic removal of bacteria [27, 40]. However, the specific role of each of these three different ubiquitin-dependent autophagy receptors and their interdependences in mediating selective engulfment of ubiquitylated bacteria, in particular with respect to hierarchical and temporal recruitment of NDP52, p62 and OPTN, remains to be determined.

 A growing body of evidence suggests that ubiquitin may serve as a general recognition signal for many targets of selective autophagy and that p62 acts as a universal receptor for this ubiquitylated cargo. Besides misfolded proteins and bacteria, p62 has been implicated in ubiquitin-dependent autophagosomal degradation of soluble proteins, peroxisome, mitochondria, and midbody ring structure [22, 41–43]. Thus, whereas p62 participates as ubiquitin receptor in many autophagic processes, NBR1, NDP52, and OPTN are specialized to function in specific types of selective autophagy. Clearly, the molecular underpinnings of this partitioning need to be mechanistically dissected in more detail. Given the plethora of ubiquitin binding domains, it would not be surprising to see more been involved in selective autophagy. A yet new twist to the autophagy receptors emerged from the identification of an NBR1-fold domain in ATG19p [9], raising the question whether some of these autophagy receptors (or at least NBR1) have ubiquitin-independent roles in targeting substrates.

## 5. Transmembrane Domain

 The targeted removal of damaged mitochondria by the autophagic machinery represents the currently best-studied example of selective autophagy of organelles that is mediated by specific autophagy adaptors. Mitochondria are the powerhouse of the cell that play a crucial role in the regulation of cellular bioenergetics and metabolism. Therefore, the maintenance of a pool of functional mitochondria is vital for the cellular homeostasis. NIP3-like protein X (Nix), which is also known as BCL2/adenovirus E1B 19 kDa interacting protein 3-like (BNIP3L) (Figure 1(d)), was cloned back in 1998 via its homology with Bnip3 from a human placenta cDNA library [44]. Under physiological conditions, Nix localizes to the mitochondrial outer membrane, where it is anchored via its transmembrane domain. Nix functions as a mitophagy receptor in mammalian cells that mediates selective clearance of mitochondria [45]. The phenotype of Nix-deficient mice is characterized by defective erythrocyte differentiation with high reticulocyte count and corresponding anemia. This phenotype is due to impaired removal of mitochondria from reticulocytes by mitophagy due to the failure to deliver damaged mitochondria to autophagosomes. Removal of mitochondria from reticulocytes represents a prototype form of programmed mitophagy in development and is a crucial step during erythropoiesis for the proper differentiation of erythrocytes that normally become devoid of mitochondria once they pass the reticulocyte status [46, 47]. Nix expression becomes markedly upregulated during the terminal differentiation stages of red blood cells [48], in line with its key role in the programmed removal of mitochondria during development.

 In addition, Nix has been implied to mediate the ubiquitylation of damaged mitochondria by the E3 ligase Parkin [49]. Upon depolarization of mitochondria, which marks an early step of mitochondrial dysfunction, Nix facilitates the recruitment of Parkin to depolarized mitochondria [49]. In addition, Pink1 is required for Parkin recruitment to mitochondria [50]. Parkin in turn labels mitochondria for the removal by the autophagic machinery through ubiquitylation of mitochondrial proteins such as VDAC1 and mitofusins [22, 51, 52]. However, additional studies are required to determine what the ubiquitylated target molecules are on the mitochondrial membranes that mediate the autophagic clearance of mitochondria during mitophagy. Moreover, Nix may also initiate mitophagy by causing mitochondrial depolarization, as Nix is also an inducer of mitochondrial cell death [53].

 Bnip3 was originally identified as interaction partner of Bcl-2 and adenovirus E1B 19 kDa protein in a yeast two-hybrid screen [54]. Based on the homology to Nix, Bnip3 is likely an additional mitophagy receptor. Bnip3 is anchored to mitochondria via its C-terminal transmembrane domain [53]. Bnip3-mediated mitophagy is triggered upon hypoxia as part of an adaptive, HIF1-dependent response [55]. Since Nix is also induced by hypoxia [56], Bnip3 and Nix may have overlapping functions. Also, Bnip3 and Nix may have a broader role in the regulation of hypoxia-triggered autophagy by interfering with the Bcl-2/Beclin-1 interaction via their BH3 domain, which in turn results in the activation of bulk autophagy by stimulating the Beclin-1/class II PI3K complex [56].

 A similar control of mitophagy by selective autophagy receptors exists also in the yeast system. There, Atg32 represents the mitophagy receptor that resides in the mitochondrial outer membrane (Figure 1(d)) [57, 58].

## 6. Autophagosomes Recruitment Motifs in Autophagy Receptors

**Figure 2:**
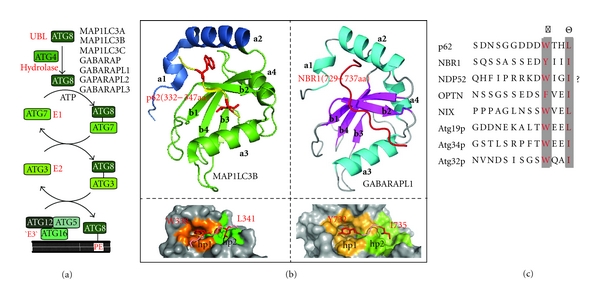
(a) ATG8 conjugation cascade. (b) Structures of MAP1LC3B/p62-LIR (upper left; pdb code: 2K6Q) and GABARAPL1/NBR1-LIR (upper right; pdb code: 2ZJD) complexes. LIR binding sites of MAP1LC3B (lower left) and GABARAPL1 (lower right). (c) Sequence alignments of functional LIR motifs in autophagy receptors.

 Once cargo destined for selective autophagy is bound by the respective autophagy receptors, subsequent delivery to the autophagosomal membrane is mediated by interaction between cargo-specific autophagy receptor proteins and members of the ATG8 ubiquitin-like (Ubl) protein family (Figure 1(b)). The evolutionary conserved ATG8 family encompasses Atg8p in yeast and seven members in humans (microtubule-associated protein-1 light chain 3A (MAP1LC3A), MAP1LC3B, MAP1LC3C, *γ*-aminobutyric acid type A (GABA) receptor-associated protein (GABARAP), GABARAP-like 1 (GABARAPL1), GABARAPL2, and GABARAPL3) [59, 60]. ATG8 is unconventionally conjugated to phosphatidylethanolamine (PE) via its C-terminal Glycine residue through the action of an E1-E2-E3 conjugation cascade involving ATG7, ATG3, and the oligomeric complex formed by ATG16-ATG5–ATG12 (where – refers to a covalent bond) (Figure 2(a)) [61–64].

 Lipidated ATG8 is thereby incorporated into the membrane of the developing autophagosome and serves as docking site for specific autophagy adaptors. The direct interaction between lipidated ATG8 and autophagy adaptors tethers cargo specifically bound by distinct adaptors to the site of autophagosome formation, leading to engulfment and sequestration of ATG8-adaptor-cargo complexes in autophagosomes. The structural basis of adaptor docking to ATG8 has been revealed by several analyses [65–68]. Briefly, ATG8 proteins generally adopt an ubiquitin fold with an N-terminal extension encompassing two *α*-helices (*α*1 and *α*2). An exposed *β*-strand (*β*2) within the ubiquitin fold of ATG8 and two adjacent hydrophobic pockets (hp1 and hp2), formed mainly by residues originating from *β*1, *β*2, and *α*3 critically, contributes to adaptor protein binding (Figure 2(b)).

 Importantly, this docking site is conserved among different ATG8 family members. Though structurally divert, all known autophagy adaptors (Atg19, Atg34, p62, NBR1, NDP52, OPTN, Nix, and ATG32) harbor a common, short linear peptide motif, which binds to the ATG8-docking site and thereby essentially mediates direct adaptor-ATG8 interaction (Figure 2(c)) [69]. Note that the LIR in NDP52 is only a candidate LIR motif based on bioinformatics studies and has not been confirmed experimentally. Due to its initial identification in the context of MAP1LC3B (LC3) binding [30, 65], this peptide motif was paradigmatically termed LC3-interaction region (LIR). The consensus sequence for the LIR motif is broadly defined as ΘxxΓ wherein Θ and Γ represent aromatic (i.e., tryptophan, tyrosine, and phenylalanine) and hydrophobic (i.e., leucine and Isoleucine) residues, respectively (Figure 2(b) and 2(c)) [68]. Residues at the Θ position bind to ATG8‘s hp1, whereas residues at the Γ position bind hp2. LIR peptides adopt an extended *β* conformation and form an intermolecular *β*-sheet with *β*2 of ATG8. Notably, acidic residues N-terminally preceding the LIR motif have been shown to additionally contribute to the LIR-ATG8 interaction, possibly by interacting with the positively charged *α*2 [68]. Recent NMR studies revealed that tryptophan in the Γ position has the strongest influence on binding affinities [68]. Remarkably, mutation of a single residue at the Γ position within the LIR motif of p62 (W338A), OPTN (F178A), Nix (W35A) or Atg19p (W412A) abrogated binding to ATG8/LC3/GABARAP proteins [27, 30, 45, 66, 70]. As a functional consequence, these autophagy receptors retain binding to their respective cargo but fail to be recruited into autophagosomes. For example, OPTN carrying the LIR mutant F178A was detected on *Salmonella* but was unable to restrict bacterial growth upon gene complementation in cells, underlining the functional role of OPTN as autophagy receptor in recruiting *Salmonella* into autophagosomes for their degradation [27].

 Lastly, LIR motifs are not restricted to autophagy receptors but emerge as a general surface for interaction with ATG8 family proteins. For example, functional LIR motifs have been identified in adaptor proteins regulating movement of autophagosomes along microtubule and autophagosome maturation such as the Rab7 effector FYCO1 and TBC domain-containing GTPase-activating protein TBC1D25, respectively, as well as components of the ATG8 conjugation system such as ATG3 [71–73]. A recent proteomic approach coupled to *in vitro* binding studies identified numerous proteins as novel ATG8-binding proteins [74]. Despite challenging due to the shortness of LIR motifs, a systematical bioinformatics-based identification of candidate LIR motifs followed by their experimental validation will be critical to assess the cellular repertoire of autophagy receptors and other regulatory ATG8-interacting proteins.

## 7. Regulation of Cargo-Receptor-ATG8 Complex Assembly

 Until lately, the spatiotemporal regulation of cargo binding by autophagy receptors and the subsequent recruitment of cargo-receptor complexes to autophagic membranes for selective engulfment remained elusive. Recent reports by two different groups have now shed light on possible mechanisms controlling dynamics of cargo-receptor and receptor-ATG8 interactions, respectively. First, p62 is specifically phosphorylated at serine 403 (S403), which resides within its UBA domain [75]. S403 phosphorylation increases the affinity between UBA and polyubiquitin chain. Intriguingly, upon binding of phosphorylated UBA to a polyubiquitin chain, phospho-S403 is not accessible for dephosphorylation anymore, indicating a possible mechanism for capturing ubiquitylated proteins for formation of aggregates and autophagosomal engulfment, respectively. These findings raise the questions whether other ubiquitin-binding domains are similarly regulated by phosphorylation. Casein kinase 2 (CK2) has been demonstrated to phosphorylate S403 of p62 directly *in vitro *and in cells. However, determining the kinase network responsible for ubiquitin-binding inducing phosphorylation events will be critical for understanding the signaling circuits underlying cargo binding via ubiquitin-dependent autophagy receptors in particular and ubiquitin-binding proteins in general.

 Second, recognition of bacterial pathogens in the cytosol through specific pattern-recognition receptors as part of the innate immune response eventually leads to activation of TANK-binding kinase 1 (TBK1), which in turn binds and phosphorylates OPTN at a serine residue (S177) that precedes the hydrophobic core sequence of the LIR motif in OPTN [27]. S177 phosphorylation causes an increase in the affinity of OPTN for MAP1LC3B. Mechanistically, the increase in binding affinity due to the presence of the phosphoserine preceding OPTN's LIR motif might result in altered hydrogen bond formation, which could potentially counterbalance the suboptimal binding affinity of the unmodified LIR sequence context due to the presence of phenylalanine instead of tryptophan at the Γ position within the LIR motif of OPTN. Remarkably, a phospho-mimicking version of OPTN bound to MAP1LC3B with a higher affinity than its wild-type counterpart, while a nonphosphorylatable version of the protein was strongly impaired in its MAP1LC3B-binding ability. Thus, recruitment of TBK1 and OPTN to the surface of ubiquitylated Salmonella leads to spatial activation of TBK1 to enable timely recruitment of MAP1LC3 by OPTN. As mentioned above, TBK1 recruitment is mediated by NDP52, placing the autophagy receptor NDP52 potentially upstream of TBK1 and OPTN. However, the hierarchical nature of signaling events leading to autophagosomal engulfment of ubiquitylated bacteria is still poorly understood. Furthermore, whether conserved serine residues preceding the LIR motifs of Nix and NBR1 are phosphorylated to control autophagosomal engulfment similarly to OPTN remains to be addressed.

 Finally, several adaptor proteins facilitating cargo-receptor-ATG8 assembly on incipient autophagic membranes are implicated in selective autophagy, though their specific functions are not well characterized yet. In yeast, Atg11p acts as an adaptor protein for Atg19p, Atg34p, and Atg32p [10–12, 57, 58]. Atg11p binds directly to Atg19p, Atg34p, and Atg32p and is responsible for recruitment of receptor-cargo complexes to the PAS for autophagosomal engulfment via interaction with Atg1p and Atg17p. In mammals, the 400 kDa scaffold autophagy-linked FYVE protein (ALFY) has been implicated in selective autophagy [76]. ALFY translocates from the nucleus or nuclear envelope to autophagic structures in the cytosol in response to amino acid starvation and binds p62 via a C-terminal BEACH domain. Additionally, ALFY binds ATG5 via a WD40 repeat region and PtdIns(3)P through a FYVE domain [76, 77]. Similar to p62 and NBR1, ALFY is required to recruit ubiquitylated proteins into aggregates prior to their autophagosomal degradation [78]. Intriguingly, deletion of the ALFY homologue in flies led to accumulation of ubiquitylated protein aggregates and manifestation of a neurodegenerative phenotype [79]. A common feature of these two structurally diverse adaptor proteins seems to be their ability to tether cargo-receptor complexes to autophagic membranes, thereby mediating recruitment to the site of autophagosomal engulfment. As a functional consequence, adaptor proteins might ensure that cargo-receptor complexes only bind to ATG8 proteins lipidated to autophagic membranes and prevent presumably unproductive interactions with cytosolic, free forms of ATG8 proteins.

## 8. Concluding Remarks

The work described here underscores the mechanistic and architectural complexities employed in selective autophagy to control autophagosomal turnover of a broad range of selective substrates ranging from proteins, via organelles to whole organisms. However, many questions remain concerning identities of additional cargo and receptor pairs as well as signaling cascades leading to efficient cargo binding and recruitment to autophagic membranes under different physiological and pathophysiological conditions.
